# Albany City Hospital

**Published:** 1870-02

**Authors:** 


### Albany City Hospital.

It appears that Dr. Charles A. Robertson's review of the published report of the case of Prof. March, has obtained his dismissal from the (so called) Eye and Ear Department of the Albany City Hospital. The action of the Governors so far as can be known from the present statement of facts, appears quite inconsistent with fairness and a true sense of their obligations to the public. Again, the public expose of the manner in which the Hospital obtained $4,000, appropriated by the State for the Eye and Ear Hospital, is not very complimentary to their own honesty. Dr. Robertson is too sharp and too much in the right to be treated unhandsomely with safety, and we believe his dismissal from the Hospital will return to torment his pursuers. Certainly if he has been treated as now appears, unfairly, it will only advance his own name and standing, and work irreparable injury to the Hospital and the men who have effected his removal.

